# Early-onset esophageal squamous cell carcinoma with achalasia: A case report

**DOI:** 10.1097/MD.0000000000037140

**Published:** 2023-02-02

**Authors:** Shujin Li, Xin Chen, Lili Zhang, Hong Jin, Bin Wang, Cong Liu, Shiwei Ru, Xuechai Liu, Wei Zhao

**Affiliations:** a Department of Gastroenterology and Hepatology, General Hospital of Tianjin Medical University, Tianjin, China.

**Keywords:** achalasia, cancer early detection, esophageal squamous cell carcinoma, medical family history, per-oral endoscopic myotomy

## Abstract

**Rationale:**

Individuals afflicted with achalasia of the cardia (AC) are more susceptible to the development of esophageal cancer (EC). However, the presence of esophageal retention obscured observation, making it difficult to detect EC early, which leads to misdiagnosis and poor prognosis in AC patients with EC. Besides, the persistence of high-risk factors may have contributed to the rapid progression of EC shortly after per-oral endoscopic myotomy (POEM). Therefore, it is imperative to alert clinicians to this extremely rare and instructive early-onset cancer.

**Patient concerns:**

The patient was a 67-year-old male who developed dysphagia 3 years ago without obvious causes, with intermittent onset and aggravating trend, accompanied by weight loss. He usually eats high-temperature foods and pickled foods, and has a family history of esophageal squamous cell carcinoma.

**Diagnosis and interventions:**

The patient was initially diagnosed with AC 2 years ago and subsequently underwent POEM surgery. One year after surgery, he was found to have mid-upper EC during follow-up and underwent partial esophagectomy in time.

**Outcomes:**

The patient’s symptoms have significantly improved with weight gain, and he is still adhering to regular follow-up and endoscopic examination.

**Lessons:**

In rare cases, EC develops early in patients with achalasia after POEM surgery. To avoid missed diagnosis, a comprehensive examination to improve the accuracy to diagnose achalasia and identify possible early-onset cancer is very important in clinical practice. Especially for patients with AC who have a family history of EC or other high-risk factors may develop EC early after POEM surgery. Therefore, regular endoscopic follow-up after POEM surgery is essential.

## 1. Introduction

Achalasia of the cardia (AC) is a primary esophageal motility disorder of unknown origin, characterized by a relaxation disturbance in the lower sphincter of the esophagus and loss of peristalsis in the esophagus. The main pathophysiological mechanism described is a decrease in the number of myenteric neurons, or their absence, causing aperistalsis and impaired relaxation of the lower esophageal sphincter. Most likely, the myenteric neurons disappear due to chronic ganglionitis.^[1]^ These abnormalities lead to impaired emptying of food from the esophagus into the stomach with resulting food stasis. Consequently, most patients experience severe dysphagia as well as other complications, such as megaesophagus,^[2]^ aspiration pneumonia,^[3]^ and esophageal cancer, specifically esophageal squamous cell carcinoma (ESCC).^[4,5]^ However, in the pathogenesis of ESCC, the treatments performed for achalasia (such as balloon dilatation, Heller myotomy, per-oral endoscopic myotomy (POEM), and botulinum injection) lowering the lower esophageal sphincter (LES) pressure and relieving the patients’ symptoms, are not effective in restoring esophageal peristalsis. The present article reports a case of early development of ESCC in a patient with achalasia with a family history of tumors, aiming to remind clinicians to pay attention to careful examination at the time of diagnosis, and should not ignore the possible potential pathogenic role of high-risk factors such as tumor family history.

## 2. Case presentation

**Figure 1.**
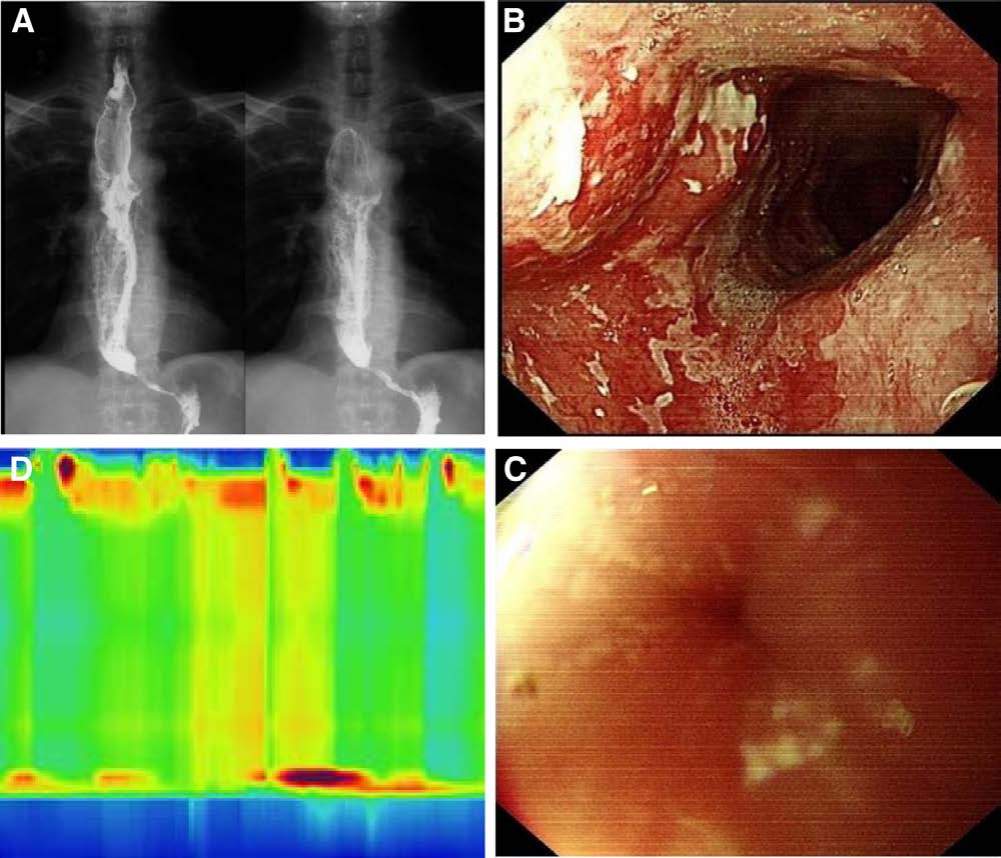
The results of upper gastrointestinal angiography, gastroscopy, and high resolution esophageal manometry in diagnosis of achalasia. (A) Upper gastrointestinal angiography showing enlarged esophageal lumen and narrowing of the lumens at the cardia, revealing a “bird’s beak sign”. (B) Gastroscopy suggesting the presence of an extensive white plaque coating the mucosal lining within the mid-esophagus. (C) EGD encountered resistance at LES and the cardia was tightly closed. (D) High-resolution esophageal manometry assessments indicating impairment in LES relaxation and a notable absence of peristaltic activity in the esophageal body. EGD = esophagogastroduodenoscopy, LES = lower esophageal sphincter.

**Figure 2.**
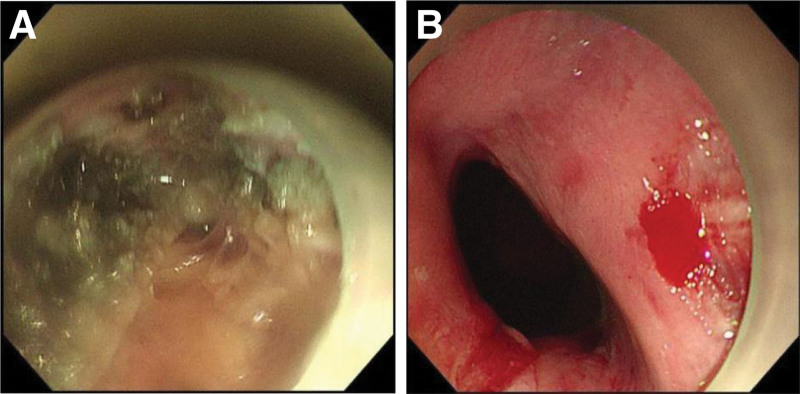
Endoscopic observation during POEM. (A) During POEM surgery, the muscular layer of the esophagus is incised under gastroscopy. (B) Postoperative POEM can be seen with the opening of the cardia. POEM = per-oral endoscopic myotomy.

**Figure 3.**
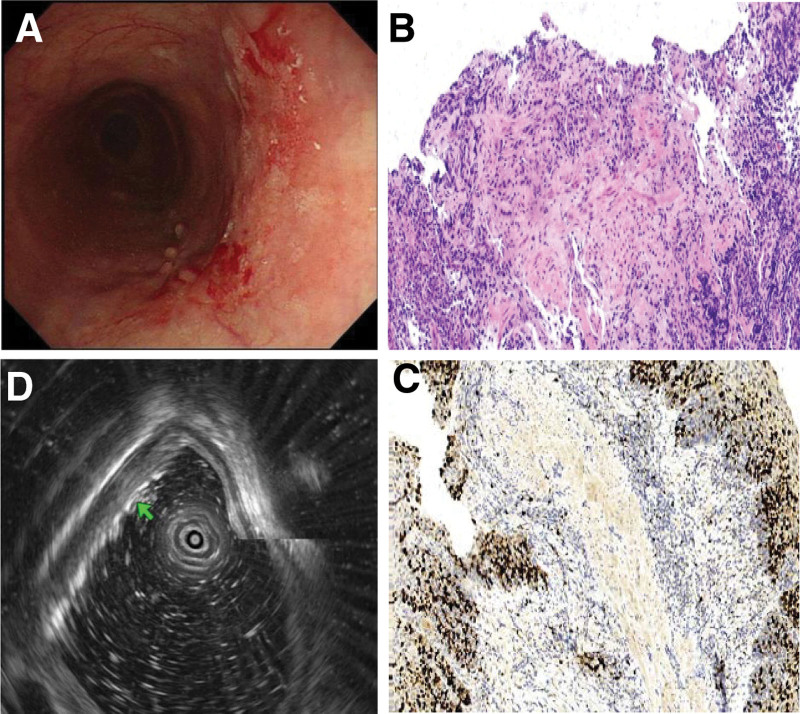
The results of gastroscopy, ultrasound gastroscopy, and immunohistochemical staining of biopsy (400×) during postoperative follow-up. (A) Gastroscopy showing the presence of irregular circumferential mucosal alterations characterized by erythematous areas, roughness, and adherent opaque substances located at a distance of approximately 21 to 31 cm from the incisors. (B) Biopsy pathology showing high-grade squamous intraepithelial neoplasia, accompanied by regions displaying in situ carcinoma. (C) Immunohistochemistry staining revealing the Ki-67 proliferation index of approximately 40%. (D) Ultrasound gastroscopy showing the mucosal layer at the lesion was slightly thicker, and the 3 layers of mucosa, muscularis mucosae, and submucosa were not clearly demarcated, but the integrity of the muscularis propria remained preserved.

The patient was a 67-year-old male who initially presented with pronounced difficulty in swallowing solid or dry food items 3 years ago. Over the subsequent months, dysphagia was observed intermittently and was often accompanied by reflux episodes that occurred several hours after meals. Notably, the reflux material consisted of undigested food and lacked gastric acid or bile components. As the patient’s symptoms progressively worsened, there was a noticeable decline in body weight. It is worth mentioning that the patient exhibited a preference for consuming foods with elevated temperatures and salt content and had a documented family history of ESCC. Approximately 2 years ago, the patient underwent esophageal imaging at a local medical facility, which revealed the characteristic “bird’s beak” sign (Fig. 1A). Subsequent esophagogastroduodenoscopy (EGD) unveiled esophageal dilation, evident food retention, and the presence of an extensive white plaque coating the mucosal lining within the mid-esophagus (Fig. 1B). EGD further encountered resistance at the LES (Fig. 1C). High-resolution esophageal manometry assessments indicated impairment in LES relaxation and a notable absence of peristaltic activity in the esophageal body (Fig. 1D), ultimately culminating in a diagnosis of AC. Following this diagnosis, the patient underwent a therapeutic intervention in the form of POEM (Fig. 2), which yielded a significant amelioration of symptoms, as reflected by a decline in the Eckardt score from 6 to 2, along with weight gain. Subsequent to a year post-POEM, the patient sought medical care at our institution for scheduled follow-up. During this examination, EGD demonstrated a reduction in the degree of esophageal dilation, the presence of salivary retention, and the identification of irregular circumferential mucosal alterations characterized by erythematous areas, roughness, and adherent opaque substances located at a distance of approximately 21 to 31 cm from the incisors (Fig. 3A). This endoscopic presentation corresponded to a 0-IIb lesion featuring localized granular changes. Application of magnifying endoscopy with narrow-band imaging revealed a discernible demarcation line (+), primarily exhibiting type B1 patterns, with some instances of B2, and showcasing a diffuse irregular microvascular architecture (S). Lugol iodine staining failed to depict any iodine uptake, pink sign (+), silver-stained sign (+), and Tatami-me’ sign (−). Multiple biopsy specimens were acquired and subjected to histopathological analysis, which conclusively confirmed the presence of high-grade squamous intraepithelial neoplasia, accompanied by regions displaying in situ carcinoma (Fig. 3B). Immunohistochemistry staining revealed positive staining for cytokeratin in the neoplastic cells, coupled with a Ki-67 proliferation index of approximately 40% (Fig. 3C). Endoscopic ultrasound assessments indicated mucosal edema, roughness, and erosions affecting approximately one-half to one-third of the esophageal lumen circumference, located 20–30 cm from the incisors. Notably, the mucosal layer exhibited mild thickening at the site of the lesion, with an indistinct demarcation between the mucosa, muscularis mucosae and submucosa; however, the integrity of the muscularis propria remained preserved (Fig. 3D). In the subsequent course of clinical evaluation, the patient received a definitive diagnosis of ESCC that had developed in the post-POEM state of AC. Subsequently, the patient underwent a surgical intervention involving thoracoabdominal laparoscopy combined with cervicothoracoabdominal 3-incision esophageal resection surgery. Intraoperatively, the malignancy was identified within the upper-middle segment of the esophagus, spanning approximately 10 cm. Postoperative pathological analysis confirmed the presence of ESCC characterized by moderate to poor differentiation and invasion into the submucosal layer of the esophageal wall. Immunohistochemistry results indicated positivity for P40, P63, and Ber-EP4 (partial) in the cancerous cells, while CK7 staining was negative. The Ki-67 proliferation index was estimated to be approximately 40%. During regular follow-up of the patient 1 year after surgery, we found that the patient’s clinical symptoms have significantly alleviated without recurrence, accompanied by weight gain. At present, the patient still adheres to regular follow-up and endoscopic examination.

## 3. Discussion

AC represents a rare primary esophageal motility disorder with an annual incidence of approximately 1 in 100,000. Recent years have seen a rising trend in its occurrence. The predominant clinical symptom is dysphagia, which tends to worsen as the condition progresses, leading to compensatory esophageal dilation in AC patients. This prolonged retention of food and liquids, coupled with bacterial overgrowth and impaired gastric acid clearance, can result in chronic inflammation, epithelial hyperplasia, multifocal dysplasia, and, in rare cases, ESCC in the esophageal mucosa.^[6–8]^

Remarkably, research^[5]^ from Germany has revealed that AC patients face a significantly higher risk (140-fold) of developing esophageal carcinoma (EC) compared to the general population (which has an annual incidence of 4 per 100,000). Some reports suggest that the probability of EC in AC patients surpasses even that of high-incidence EC regions by more than tenfold. Therefore, AC patients are reasonably considered a high-risk group for EC, and the etiology appears to be linked to esophageal stasis.

Earlier studies have indicated that EC may manifest in AC patients many years after the onset of AC symptoms, typically emerging 20 to 25 years post-symptom debut. EC can also occur after Heller myotomy or balloon dilation, albeit at a lower rate (1.6%–2.7%).^[9–11]^ However, the patient detailed in this article developed ESCC a mere 3 years after the onset of dysphagia symptoms, a markedly shorter timeframe than previously reported. We speculate that the patient’s unhealthy dietary habits and family history of tumors may have played significant roles in this accelerated progression.

The pathological type of EC in AC patients is predominantly ESCC (74.5%),^[11]^ with adenocarcinoma and rare esophageal sarcomas also reported.^[12]^ The most common site of lesions is the middle segment of the esophagus, followed by the lower segment,^[11]^ although there are also reports of upper esophageal cancer.^[13]^ In this case, the patient had ESCC in the upper-middle segment of the esophagus, consistent with previous statistical results.

Currently, the chronological relationship between ESCC onset and the timing of POEM remains unclear. It is possible that the patient already harbored ESCC before the POEM procedure. Early-stage ESCC often lacks hallmark symptoms of progressive dysphagia seen in EC, making it challenging to differentiate from AC symptoms. Furthermore, the unaffected lower esophageal segment maintains mucosal smoothness, resulting in the persistent appearance of the characteristic “bird’s beak” sign during esophagography, which may explain the delayed ESCC detection in this case. Additionally, the patient exhibited esophageal retention, mucosal edema, and mucous adherence before POEM, which restricted the field of view, potentially masking the lesion and increasing the difficulty of early esophageal carcinoma detection. Alternatively, it cannot be ruled out that the patient developed esophageal cancer one year post-POEM.

Reports have suggested that POEM treatment, as a primary AC therapeutic modality,^[12,14]^ may reduce the risk of EC (reducing incidence to 1/8 compared to untreated individuals).^[15]^ This could be linked to improved esophageal stasis^[16]^ and a reduction in the positivity rate of Ki-67 in esophageal squamous epithelium.^[12]^ However, while POEM may delay the onset, it may not completely prevent esophageal epithelial carcinogenesis in AC patients, particularly if they have other high-risk factors such as poor dietary habits or a genetic predisposition.

Our study has several important limitations. (1) This case suggests that AC patients with a family history of esophageal cancer are more likely to develop early esophageal cancer after POEM, but there is a lack of evidence from multi-center prospective studies comparing the incidence and timing of esophageal cancer after POEM in AC patients with or without a family history of cancer. (2) At present, the patient in this case has only been followed up for 1 year after surgery, and the clinical symptoms have significantly improved without recurrence. However, the long-term efficacy of treatment and the possibility of recurrence still require further follow-up observation.

## 4. Conclusion

In summary, this article highlights a case of early detection of ESCC in a patient with a family history of EC after POEM. AC is considered a premalignant condition,^[9]^ and the coexistence of AC and EC can lead to misdiagnosis and poor prognosis. Therefore, diagnosing AC requires a comprehensive approach involving endoscopy, high-resolution esophageal manometry, and esophagography. Attention should be paid not only to excluding cardia fundic carcinoma but also to the possibility of esophageal mucosal carcinogenesis in AC patients. Adequate esophageal cavity lavage before examination is essential to optimize mucosal exposure and minimize the risk of missed diagnoses. When necessary, a combination of examination techniques can be used to assess lesion depth and assist in formulating a treatment plan.^[17,18]^ It is crucial to emphasize that AC patients with a family history of EC or poor dietary habits should prioritize regular post-POEM endoscopic follow-up (following EC screening criteria) to facilitate early EC detection.^[19,20]^

## Acknowledgments

We would like to thank the patient and his family.

## Author contributions

**Conceptualization:** Shujin Li, Lili Zhang, Hong Jin, Bin Wang.

**Data curation:** Shujin Li, Cong Liu, Shiwei Ru, Xuechai Liu.

**Supervision:** Xin Chen, Wei Zhao.

**Writing – original draft:** Shujin Li.

**Writing – review & editing:** Wei Zhao.
